# *In vitro* PK/PD modeling and simulation to accurately assess the antimicrobial activity of tigecycline against *Mycobacterium abscessus*

**DOI:** 10.1128/aac.01025-25

**Published:** 2025-12-23

**Authors:** Hyunseo Park, Sara E. Maloney Norcross, Anthony J. Hickey, Mercedes Gonzalez-Juarrero, Bernd Meibohm

**Affiliations:** 1 Department of Pharmaceutical Sciences, College of Pharmacy, University of Tennessee Health Science Center 12326, Memphis, Tennessee, USA; 2 Technology Advancement and Commercialization, RTI International 6856, Durham, North Carolina, USA; 3 Mycobacteria Research Laboratories, Department of Microbiology, Immunology and Pathology, Colorado State University 3447, Fort Collins, Colorado, USA; City St George's, University of London, London, United Kingdom

**Keywords:** *Mycobacterium abscessus*, non-tuberculous mycobacteria, tigecycline, PK/PD

## Abstract

Conventional *in vitro* susceptibility testing methods may underestimate the bactericidal activity of antibiotics that are chemically unstable in aqueous media, thereby limiting their clinical translatability. Tigecycline is considered a representative example of such compounds, exhibiting notable therapeutic efficacy against a broad range of pathogens despite poor *in vitro* susceptibility profiles, as reflected by elevated MIC values. This discrepancy is likely attributable, at least in part, to the chemical instability of TGC under standard MIC assay conditions. In this manuscript, we propose a mechanism-based PK/PD modeling approach as a framework to overcome the limitations of traditional MIC assessments and to address potential discrepancies between intrinsic and experimentally measured apparent antibacterial activity. Dynamic time-kill curves for single and multiple dose scenarios of TGC against *Mycobacterium abscessus (Mab*) were experimentally simulated in 24-well plates, leveraging the chemical instability of TGC. Based on the resulting *in vitro* data, a mechanism-based model was developed to perform simulations for characterizing intrinsic efficacy and potency of TGC. While the *in vitro* MIC of TGC determined under standard conditions was determined as 3.125 mg/L, an intrinsic MIC simulated based on model predicted bacterial time-time kill curves was 0.5 mg/L. Model-based analysis also revealed that MIC under standard conditions was stemming from drug instability and bacterial growth rate in the utilized media. In conclusion, the PK/PD modeling and simulation-based MIC determination indicated that clinically achievable exposure levels of TGC are sufficient to kill *Mab*, underlining the therapeutic potential of TGC against *Mab* infections.

## INTRODUCTION

Nontuberculous mycobacteria (NTM) are ubiquitous commensal organisms increasingly recognized as human opportunistic pathogens with a rising incidence of infections (1). *Mycobacterium abscessus (Mab*) is classified as a rapidly growing NTM and is not limited to but frequently responsible for pulmonary infections, especially in cystic fibrosis patients (2, 3). With an average treatment success rate of only 45% for antibiotic interventions based on current treatment guidelines (4), depending on the subspecies and strains (5), control of pulmonary infections caused by *Mab* remains an unmet medical need.

Tigecycline, a third-generation tetracycline derivative approved in 2005, was designed to overcome several known bacterial resistance mechanisms, such as tetracycline-specific efflux pumps and ribosomal protection, thereby conferring a broad range of antibiotic activity against numerous bacterial species (6). Early reports suggested that more than 60% of patients, including those with cystic fibrosis, showed improved clinical outcomes against *Mab* infection when TGC was given as part of a multidrug regimen to patients with prior antibiotic treatment failures (7). Intravenous TGC therapy, however, was also accompanied by a high incidence of dose-limiting gastrointestinal adverse events, particularly nausea and vomiting (7). A meta-analysis of the exposure-response relationship in multiple randomized clinical trials involving 169 patients who received the standard dosing regimen of TGC, 50 mg given intravenously twice daily, was conducted (8). The steady-state area under the plasma concentration-time curve for 24 h (AUC_0-24_)-based exposure of 6.87 mg h L^−1^ was identified as a threshold that was associated with the increased occurrence of nausea and/or vomiting. Thus, the clinically usable exposure range of TGC for treating *Mab* remains limited.

It is well known that TGC has chemical instability in aqueous solutions (9). While freshly reconstituted and diluted TGC from its commercial dosage form may be stored up to 24 h at room temperature and, thus, TGC’s limited aqueous stability has very limited impact for its clinical use, chemical degradation in aqueous media, such as Mueller-Hinton Broth (MHB) and Middlebrook 7H9 broth (7H9), during prolonged incubation for 72 h at 37°C may introduce bias during *in vitro* sensitivity (MIC) testing. Therefore, various approaches have been considered to mitigate this issue, such as using oxyrase (10) or antioxidants (9), but none has been able to completely resolve the problem. While experimental systems determining MIC are relatively simple, the data obtained are influenced by a combination of factors, including the sensitivity of the bacterial strain, the bacterial growth rate in the applied media, and the chemical stability of the tested antibiotic. If the stability of the antibiotic cannot be assumed, it becomes technically challenging to accurately assess its intrinsic potency represented by MIC as drug concentrations would vary throughout the incubation period decreasing over time. The chemical instability issue is likely more relevant for drugs that inhibit bacterial growth rather than those that exert direct bactericidal effects.

In the literature, the MIC of TGC against *Mab* has frequently been reported in the range of 3.12 to 6 mg L^−1^, which is high relative to its maximum tolerated plasma exposure (11–17). Thus, these data would suggest that the clinical activity against *Mab* achieved with the standard dosing regimen may be limited, and that higher plasma exposure levels, intolerable to humans, would be required for efficacy. However, these results contradict numerous clinical reports on the efficacy of TGC in treating *Mab* infections in patients (15).

To resolve this discrepancy, we hypothesized that the disagreement between *in vitro* predicted and actual clinical antimycobacterial activity of TGC is primarily due to the instability of TGC in the incubation media during MIC testing. In this manuscript, we propose a mechanism-based PK/PD modeling approach to overcome the methodological limitations of the existing MIC assays (18) and determine the inherent, intrinsic potency of TCG against *Mab*.

## MATERIALS AND METHODS

### Drug susceptibility testing

The MIC of TGC against *Mab* (ATCC19977; American Type Culture Collection, Manassas, VA) was determined in duplicate for each medium using the broth microdilution method with freshly prepared 7H9 w/OADC (7H9) or MHB II media (Becton Dickinson, Sparks, MD). One hundred microliters of *Mab* culture at a concentration of 1 × 10^6^ CFU/mL of *Mab,* grown in 7H9 or MHB II in the early logarithmic phase, was mixed with an equal volume of respective media containing TGC serially diluted from 100 to 0.0976 mg L^−1^ in a 96 well plate (Costar, Corning, Glendale, AZ). The plates were prepared in duplicate. One plate was used for MIC measurement and the other for the microscale dynamic time-kill assay as described below. The first plate was incubated for 72 h, followed by visual inspection and colorimetric assay. For the colorimetric assay, 30 µL resazurin dye (Sigma-Aldrich, St. Louis, MO) was added to half of the plate 48 h after the start of the incubation. The viability was estimated by measuring the metabolic activity of viable *Mab* capable of converting blue resazurin into pink resorufin using a Cytation 5 Multi Mode Reader (BioTek, Winooski, VT) with excitation at 530 nm and emission at 590 nm. No spectral overlap was observed, and background subtraction was performed using media controls. The MIC was determined by the lowest TGC concentration that prevented bacterial growth assessed by visual inspection and inhibited more than 90% of resazurin metabolic conversion.

### TGC stability test in incubation media

Solutions of 3 mg L^−1^ TGC were prepared in 2 mL of 7H9 and MHB II, respectively, and incubated at 37°C for 48 h in triplicate. Samples of 50 µL were serially collected at 0, 1, 3, 7, 24, and 48 h after initiation of incubation, and TGC concentration of each sample was quantified using liquid chromatography tandem mass spectrometry (LC-MS/MS). The degradation half-life (*t*_½_) of TGC in each media was calculated based on the percentage remaining of the drug at each time point using the following equation (equation 1).


(1) $$t_{1/2\;}=ln2\times \frac{t_{2}-t_{1}}{ln\left(\frac{C_{1}}{C_{2}}\right)}=\frac{ln2\;}{K_{deg}}$$

where *t*_1_ and *t*_2_ represent the first and last observation, respectively, *C*_1_ and *C*_2_ denote the concentrations at the corresponding time points, and *K*_deg_ is the first-order degradation rate constant.

### Microscale dynamic time-kill assay

Dynamic time-kill curves for various single and multiple dose scenarios of TGC against *Mab* were experimentally simulated in a 24-well plate (Costar, Corning, Glendale, AZ) in triplicate following inoculation of 1 × 10^6^ CFU/mL of *Mab*. In this experiment, we devised the chemical degradation property of TGC in 7H9 as a driving force for the pharmacokinetics of TGC, enabling multi-level dynamic exposure against *Mab* in each well. In particular, each well was filled with 2 mL of freshly prepared 7H9 containing *Mab* grown in the logarithmic phase and treated with different TGC concentrations. Concentrated TGC was serially diluted from 4.8 to 0.075 mg mL^−1^, and 20 µL of each working solution was spiked into the well filled with 2 mL of 7H9 containing *Mab*. Accordingly, *Mab* was exposed to an initial treatment concentration range of 0.75 to 48 mg L^1^ TGC. This concentration range covered the treatment levels used in *in vitro* susceptibility tests, including MIC. Each plate was incubated at 37°C. For multiple dose scenarios, drug treatment was repeated every 24 h until day 5. Twenty microliters of sample was homogenously collected from each well immediately before drug treatment for 5 days and every consecutive day until the study was terminated. These samples were then spiked into 1,980 µL of PBS and further serially diluted if needed. One-hundred microliters of diluted samples was taken and plated on 7H10 agar plates (Becton Dickinson, Sparks, MD), and bacterial counts were enumerated after 3 days of culturing. To confirm the chemical instability-related changes of TGC concentration over time for each treatment condition in the multiple dose scenarios, a 24-well plate filled with 2 mL of 7H9 was separately prepared without *Mab* inoculation. Samples of 20 µL were collected from each well and TGC concentrations quantified using LC-MS/MS. This confirmatory assessment was performed for all seven doses and was used to calculate the TGC concentration-time profile after multiple treatments (equation 2),


(2) $$C_{t}=\frac{C_{0}\times e^{-k_{deg}\times t}\times \left(1-e^{-k_{deg}\times nd\times \tau }\right)}{\left(1-e^{-k_{deg}\times \tau }\right)}$$

where $C_{t}$ represents concentration of TGC at specific time *t*, and *C*_0_, *K*_deg_, nd, and *τ* denote initial concentration of TGC at time zero, first order degradation rate constant in 7H9, number of dose, and dosing interval, respectively.

For the microscale dynamic time-kill assay, a total of three assays were conducted with CFU assessments performed in duplicate manner. Bacterial counts and TGC concentrations at each time point were utilized in the PK/PD model development. The limit of counting was 10^2^ CFU/mL. Exposure to light as a potential source of TGC degradation was minimized on all three assays, drug susceptibility testing, stability testing in media, and microscale assay, by performing all experiments in an incubator.

### Bioanalysis of TGC in incubation media

The concentration of TGC in incubation media was quantified on an API4500 LC-MS/MS triple quadrupole mass spectrometer (AB Sciex, Foster City, CA) with electrospray ionization (ESI) in multiple reaction monitoring mode using the mass transfers of *m*/*z* 586.3 → 513.2 for TGC and *m*/*z* 595.5 → 514.3 for the deuterated TGC internal standard (d9-TGC), respectively. The mobile phase for LC-MS/MS quantification consisted of water and methanol/acetonitrile 75:25 (vol/vol), each containing 0.2% formic acid. The analytes were eluted on the C18 column (4.6 × 50 mm, 3.5 µm, Waters, Milford, MA) with gradient methodology. Duplicate calibration curves covering the observed concentration range were used to quantify concentrations of each sample, and each analytical run was validated with five different concentrations of TGC spiked into incubation media, scattered randomly between unknown sample runs. The lower limit of quantification was 2.93 ng mL^−1^. The assay accuracy ranged between 88.0% and 106.8%, while the coefficient of variation for precision was less than 8.1%.

### PK/PD model development

#### Modeling and simulation

**Fig 1:**
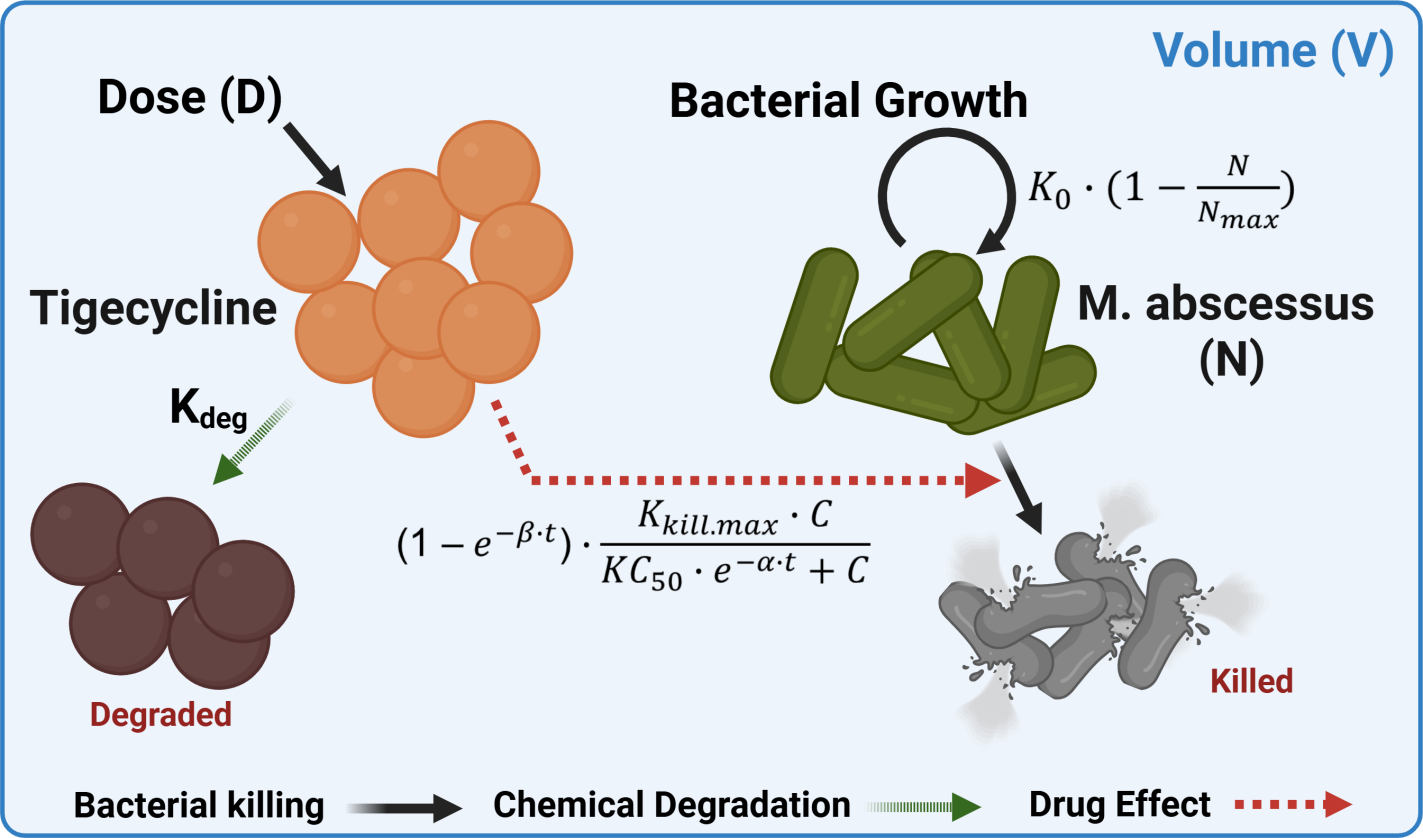
Schematic representation of the PK/PD model used to describe *in vitro* microscale dynamic time-kill assay.


Based on CFU data from the *in vitro* dynamic time-kill assay data (without log transformation), a mathematical model was developed to describe the effect of TGC on bacterial growth similar to previously used approaches (Fig. 1) (19–21).

(3) $$\frac{dC}{dt}=-K_{deg}\times \frac{Dose}{V}$$

(4) $$\frac{dN}{dt}=\left[K_{0}\times \left(1-\frac{N}{N_{max}}\right)-\left(1-e^{-\beta \times t}\right)\times \left(\frac{K_{kill.max}\times C}{KC_{50}\times e^{-\alpha \times t}+C}\right)\right]\times N$$

*K*_deg_ and *V* in equation 3 represent the TGC degradation rate constant and volume of each well, respectively. *N* in equation 4 is defined as the *Mab* count in CFU/mL, *K*_0_ as the net growth rate constant representing the difference between replication and natural death rate constant, and *N*_max_ as the maximum number of bacteria available in the system in CFU/mL. A logistic growth function, (1 – [*N*/*N*_max_]), was employed to describe the bacterial growth limit in incubation conditions without drug treatment. *K*_kill.max_ is the maximum bacterial killing rate constant, *C* is the concentration of TGC at time *t*, and KC_50_ is the concentration of TGC showing half of maximum bacterial killing efficacy of TGC in the system. Based on the observations from *in vitro* dynamic time-kill assay data, the delayed bacterial killing effect and the gradual increase in susceptibility reflected by a decrease in KC_50_ were modeled using exponential terms, with *α* and *β* as modulation rate constants, respectively. For the model, *K*_0_ and *N*_max_ are structurally identifiable from a control group or any trajectory that exhibits logistic curves. The bacterial killing terms, *K*_kill,max,_ KC_50_, *α,* and *β*, are jointly identifiable because *C*(*t*) is not a single concentration and observed for a wide range of time points with concentrations covering KC_50_. Since all experiments were densely sampled under multiple conditions, no practical identifiability issue was expected.

Random effects were considered for the initial (*N*_0_) and maximum number (*N*_max_) of bacteria in each well and for *α* and *β* to explain variation between each experimental condition and batch. Residual variability was modeled using a proportional error term:


(5) $$y_{ij}=f_{ij}\times \left(1+\varepsilon_{ij}\times \;b\right)$$

where *y*_*ij*_, *f*_*ij*_, and *ɛ*_*ij*_ represent observed data, model-based predicted outcome, and standard normal residual error following ~*N*(0,1) for the *i*th measurement at the *j*th time point, and *b* is the proportional error parameter. The model was evaluated based on parameter relevance, relative standard error (RSE), and diagnostic plots.

To predict intrinsic antimicrobial activity of TGC against *Mab*, Monte-Carlo simulations for bacterial time-kill curves were performed based on parameter estimates without drug degradation (*K*_deg_ = 0) to mimic static exposure conditions. For the determination of the intrinsic minimal inhibitory concentration (MIC_int_), independent of chemical degradation, we utilized the level of CFU/mL that does not change the color of resazurin dye after 24 h incubation as pre-defined criteria for MIC_int_ determination. All modeling and simulations were performed using nonlinear mixed effect modeling in MonolixSuite 2022R1 (Lixoft, Antony, France), with parameter estimation performed by the SAEM algorithm.

#### Model qualification

Experimental conditions derived from previously published reports for longitudinal time-kill curves of TGC against *Mabs* were compared to those used in our microscale dynamic time-kill assay in Table 1. Bacterial killing profiles were digitized from these literature sources and utilized for further evaluation and qualification of the final model and parameter estimates (12, 22, 23). Various experimental conditions, including treatment concentration, initial number of bacterial inoculations (*N*_0_), and MIC values from each paper were applied to the model as input parameters, and other parameter estimates associated with bacterial growth and killing were fixed. Time-kill curves were simulated for each experimental condition, and model performance was assessed by comparing simulated time-kill curves and their 95% confidence intervals (CI) with observed profiles reported in the corresponding references.

**TABLE 1:** Comparison of experimental conditions between the current study and literature reports for TGC and *Mabs* used for model qualification

Parameter	Current study		Pearce et al. (11)	Portell-Buj et al. (19)	Pryjma et al. (21)
Strain	ATCC19977		ATCC19977	Clinical isolates #1-#9	ATCC19977
MIC range (mg L^−1^)	3.125	0.782	4	0.125–0.5	0.8
Media	7H9	MHB II	MHB II	MHB II	MHB II
Inoculation number	5 × 10^5^		2 × 10^5^	7.08 × 10^6^	1 × 10^6^
Incubation temp. (°C)	37		30	30	37

### Model application to MIC assay platform

From the second plate of the *in vitro* drug susceptibility test, bacterial growth in freshly prepared 7H9 and MHB II under the various TGC concentration conditions was monitored in real-time using the same method described in the Microscale Dynamic Time-Kill Assay section at 0, 24, 48, and 72 h post-dose of TGC. Based on the experimental data, we modified the original model. Briefly, elimination rate constants of TGC in each media were derived and fixed as *K*_el,7H9_ and *K*_el,MHBII_, respectively, to describe the differences in stability of TGC observed. Bacterial growth rates in corresponding media were incorporated into the original model as covariates. Parameters were simultaneously estimated from dynamic time-kill curves monitored during the MIC assay performed in different media. Based on the parameter estimates, the influence of each determinant was quantitatively evaluated.

## RESULTS

### Drug susceptibility testing

The MIC of TGC obtained under the standard *in vitro* drug susceptibility test method with 7H9 media was 3.13 mg L^−1^. (Fig. S1). This value was similar to those previously reported in the literature (11–17). The MIC levels measured were higher than the exposure threshold resulting in a high incidence of gastrointestinal adverse events (7). In addition, they exceeded the peak plasma concentrations (*C*_max_) of TGC achieved after administration of single (2.82 µg mL^−1^ at 300 mg infused over 1 h) and multiple (1.17 µg mL^−1^ at 100 mg, infused over 1 h every 12 h) dose regimens in human clinical studies (24). However, the MIC determined in freshly prepared MHB II media was 0.782 µg/mL, which was fourfold lower than the 7H9-based results. This finding aligned with the bacterial killing performance of TGC in fresh MHB II media published by Amann et al. (25).

### Drug stability in incubation media

**Fig 2:**
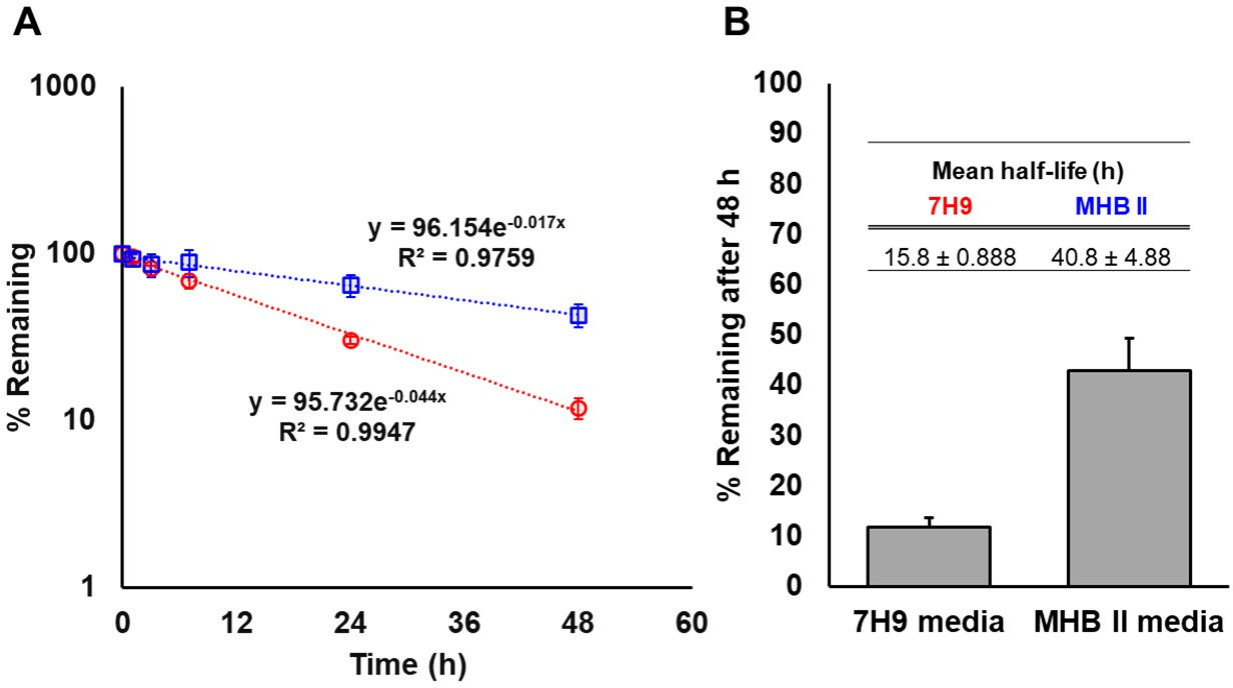
Comparison of chemical stability of TGC in 7H9 and MHB II media (*N* = 3). (**A**) First-order degradation kinetic profiles of TGC in freshly prepared 7H9 (red circles) and MHB II (blue squares) during the 48-h incubation period, and (**B**) the final percentage of TGC remaining in respective media (%) after 48 h incubation at 37°C. Error bars (±) indicate the standard deviation of the measurements.

After spiking TGC into 7H9, concentrations rapidly decreased following a first order process. Less than 20% of the original amount remained intact after 48 h incubation at 37°C, and the calculated degradation half-life was 15.8 h. No concentration-dependent differences in stability were observed (Fig. S2). On the contrary, TGC was substantially more stable when incubated at 37°C in fresh MHB II, exhibiting a 40.8 h half-life (Fig. 2). Based on the defined degradation process in 7H9, repeated addition of TGC to an incubation well resulted in a dynamically-changing concentration-time profile that is likely more biologically relevant for interactions between TGC and *Mab* than static conditions. Furthermore, the experimentally simulated multiple dose concentration-time profiles of TGC in 7H9 were well aligned with theoretically calculated concentration-time profiles based on the established degradation half-life, irrespective of the investigated dose levels (Fig. S2). The experimentally simulated TGC exposure at different levels of multiple dosing of the conducted dynamic time-kill assays was also utilized as a metric of PK/PD indices assessment. The degradation rate constant, *K*_deg_ obtained from the stability study, was employed to develop the *in vitro* PK model of TGC, under the assumption that the degradation rate remains constant regardless of bacterial growth in the media.

### Dynamic time-kill assay utilizing TGC degradation

Dynamic time-kill assays were conducted with eight different doses, as specified in the methods section, in both single and 5-days multiple dosing regimens. In control groups without TGC treatment, *Mab* grew rapidly, reached maximum CFU/mL within 96 h after inoculation and maintained the bacterial population. All treatment groups, regardless of dose, demonstrated a delayed bacterial killing effect similar to previously reported delayed time-kill kinetics of other frequently used antibiotics against *Mab* as well as rapidly growing mycobacteria (26).

**Fig 3:**
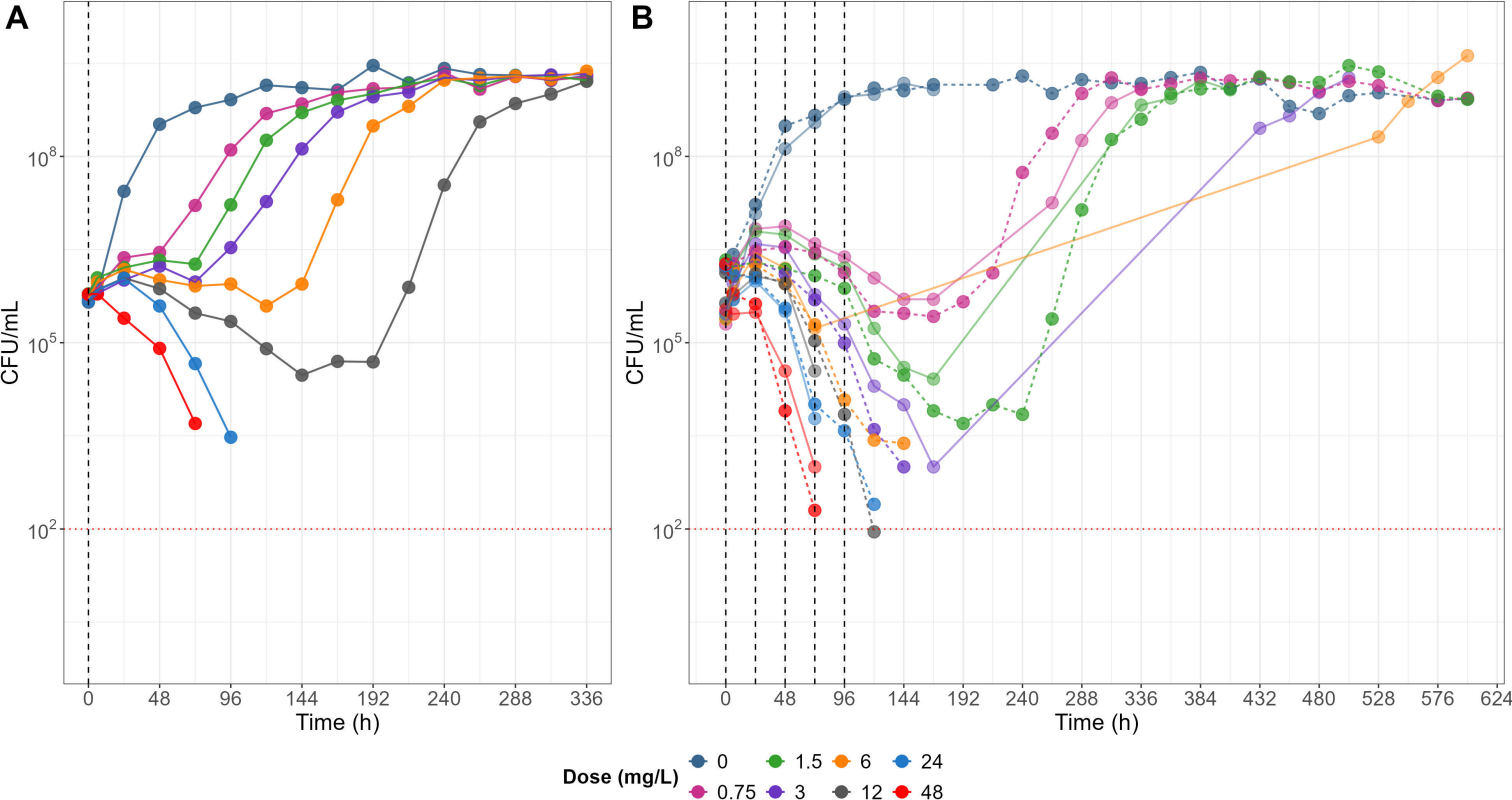
*In vitro* dynamic time-kill curves for single (**A**) and multiple (**B**) doses of TGC in the 24-well based microscale assay system. Black dashed vertical lines indicate dosing time points, and red dotted horizontal lines represent the limit of quantification (LOQ) for bacterial counts (CFU/mL). Daily dose levels (mg/L) refer to the amount of TGC (mg) added per unit volume (L) in each well.

In a single dose experiment, treatment groups that received TGC doses resulting in concentrations of 0.75, 1.5, 3, and 6 mg L^−1^ of TGC showed negligible bacterial growth until 48, 72, 96, and 120 h after TGC treatment, respectively. Following this period, the bacterial populations in those wells rapidly increased, exhibiting growth rates similar to the control group. While a weak bactericidal effect was observed in the 12 mg L^−1^ treatment group up to 144 h, bacterial regrowth occurred at 192 h post-treatment, eventually reaching the maximum number of bacterial load achievable in the 24-well-based incubating condition. However, bacterial regrowth was not observed until the end of the observation period (336 h) in groups treated with 24 mg L^−1^ or higher (Fig. 3A).

In the multiple dose experiments, a distinct bacterial killing profile was observed, especially during the initial phase, where the level of TGC in 7H9 was replenished by repeated dosing (Fig. 3B). Even at the lowest TGC dose of once daily dosing, a bacteriostatic effect was evident until the last dosing on day 5. While bacterial regrowth occurred in the low-to-intermediate dose groups after the last dose, the high dose treatment groups, including 24 and 48 mg L^−1^, did not show any regrowth until the end of the observation period (624 h), indicating a complete bactericidal effect of high dose TGC on *Mab*. The time required for bacterial regrowth post-treatment showed a positive correlation with the TGC concentrations at 24 h after the last dose of each treatment group. This was expected as the regrowth occurred when the concentration gradually decreased with first-order kinetics to below the threshold required to maintain its inhibitory effect on bacterial growth.

### PK/PD model development

The PK part of the model was described by a one-compartment model, and its parameters, volume and drug degradation rate constant, were fixed based on the experimental conditions, i.e., volume added to each well (2 mL), and degradation rate constant calculated from the drug stability test, respectively. Parameter estimates for the initial number of inoculated bacteria (*N*_0_) and maximum number of bacteria available in unit volume (*N*_max_) were 6.70 × 10^5^ and 1.40 × 10^9^ CFU/mL. A net growth rate constant (*K*_0_) of 0.12 h^−1^ and maximum bacterial killing rate constant (*K*_kill,max_) of 0.20 h^−1^ adequately described the relatively rapid growth of *Mab* (doubling time of 5.78 h in 7H9), and efficacious bacterial killing efficacy of TGC against *Mab*, respectively. The estimated KC_50_ value of 0.65 mg/L was closely aligned with the MIC value of 0.782 mg/L observed in the susceptibility study conducted using MHB II, in which TGC is relatively stable. This indicates that KC_50_ is a good indicator of the potent bacterial killing effect of TGC against *Mab*. Parameters for increase in bacterial susceptibility (*α*) and delayed bacterial killing (*β*) were 0.017 h^−1^ and 0.069 h^−1^, respectively, demonstrating that the potency and maximum killing effect increased in a time-dependent manner. Potential differences in the initial bacterial inoculation numbers among experimental groups after inoculation, along with variations in the resulting growth curves and absolute dosing amounts inherent to micro-scale studies, are likely to have influenced the initial delayed bacterial killing effect. These factors may have contributed to the relatively high inter-experiment variability observed in the associated parameters. Without incorporating these two time-dependent terms, the model did not adequately capture the overall bacterial killing dynamics of TGC (Table S1). The relative standard error (RSE) of fixed effect parameters ranged from 1.51% to 13.8% and that of random effect parameters was slightly higher but still less than 30.7%, highlighting a reasonable level of uncertainty in parameter estimates of the model. Final parameter estimates for the PK/PD model are summarized in Table 2.

**TABLE 2:** Parameter estimates of PK/PD model developed based on *in vitro* microscale dynamic time-kill assay performed in 7H9 media

Parameter	Estimate	RSE^*c*^ (%)
Fixed effects		
*V* (mL)	2.0	—^*a*^
*K*_deg_ (h^−1^)	0.041	—^*b*^
*N*_0_	6.70 × 10^5^	10.8
*K*_0_ (h^−1^)	0.12	1.83
*N*_max_	1.40 × 10^9^	6.15
*K*_kill.max_(h^−1^)	0.20	1.61
*KC*_50_ (µg mL^−1^)	0.65	1.51
α (h^−1^)	0.017	6.57
β (h^−1^)	0.069	13.8
Random effects (CV %)		
[*N*_0_]	45.0	19.6
[*N*_max_]	14.2	37.0
[α]	20.3	30.4
[β]	57.7	20.8
Residual error (proportional)		
*C*	0.19	2.77
*N*	0.47	5.43

^*a*^ Fixed based on volume per well in 24-well plate.

^*b*^ Fixed based on the results of TGC stability study in 7H9.

^*c*^ Relative standard error.

For model evaluation, diagnostic plots were utilized to assess the model predictability and potential skewness, as depicted in Fig. S3. Multiple data points indicating relationship between each observation from the dynamic time-kill assays and the corresponding model-based population predictions (Fig. S3A) and individual predictions (Fig. S3B) are evenly scattered around the identity line without significant bias. Individual weighted residuals are also randomly scattered around the horizontal zero-line, irrespective of observation time (Fig. S3C) and predicted concentration (Fig. S3D), respectively. These results confirmed that the PK/PD model developed in this study effectively captured the 24 well-based *in vitro* microscale dynamic time-kill assays of TGC performed under various experimental scenarios, including different dosing regimens.

### External model qualification

**Fig 4:**
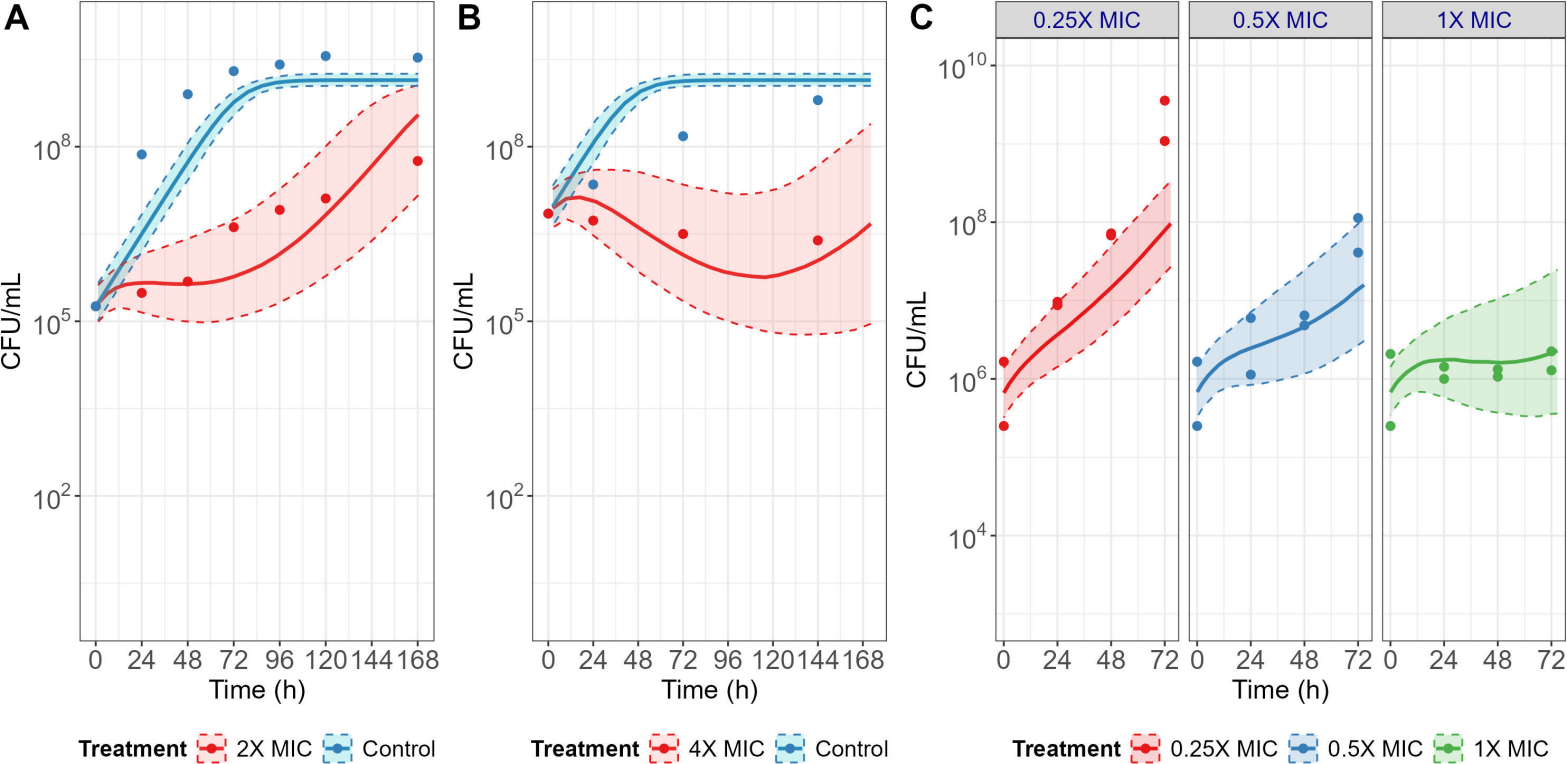
PK/PD model-simulated bacterial growth profiles under different experimental conditions (**A to C**) reported in the literature. Panels A through C correspond sequentially to the references listed in Table 1. Symbols represent observed values extracted from the literature, solid lines indicate model-based simulation profiles, and shaded areas denote the corresponding 95% confidence intervals reflecting inter-experimental variability associated with *N*_0_, *N*_max_, and bacterial killing onset. The contribution of each parameter is summarized in Table 2 as random effects.

In addition to model evaluation by assessing parameter uncertainty and diagnostic plots, diverse data sets from *in vitro* static time-kill assays performed under various experimental conditions were employed to qualify the model (Table 1) (12, 22, 23). The longitudinal time-kill curves, reconstructed by digitization from the literature and plotted in Fig. 4, showed a negligible bacterial killing effect of TGC against *Mab* except when the initial concentration of TGC was higher than eightfold MIC. These findings are aligned with the result of our 24-well-based microscale *in vitro* time-kill assay conducted with single dose without drug replenishment that exhibited a bacterial killing effect from the 24 mg L^−1^ dose group and higher. Importantly, when comparing the plots of external data sets to the model-based prediction plots using the PK/PD model and the respective conditions and input parameters of each individual paper, it revealed that the simulated time-kill curves and their 95% CI were able to adequately capture the data sets reported in the multiple literature references as depicted in Fig. 4. This suggests that the model could account for differences in experimental conditions such as *Mab* strains, stability of TGC associated with the type of media and storage, total incubation time, sampling volume, and incubation temperature (25) and further underlines its robustness.

### Simulation-based assessment of the intrinsic antibacterial potency of TGC

**Fig 5:**
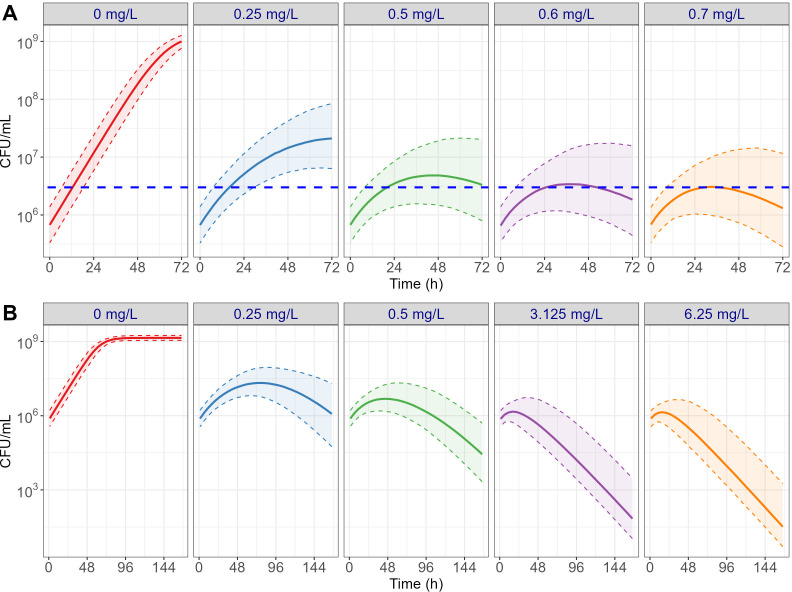
PK/PD model-based simulations of longitudinal bacterial killing profiles under the static exposure condition at various concentrations to determine MIC_int_ (**A**) and assess bactericidal effect of TGC against *Mab* (**B**). Solid lines represent model-simulation profiles, shaded areas indicate the corresponding 95% confidence intervals, and the blue dotted horizontal line denotes the MIC determination criteria.

After the model qualification steps using external data sources, the bacterial killing effect of TGC in various treatment regimens was simulated to examine MIC_int_ of TGC under static conditions without degradation, which is only feasible in a simulation setting. As shown in Fig. 5A, model-based simulations for time-kill curves were conducted up to the incubation time typically required for standard drug susceptibility testing of rapidly growing mycobacteria (72 h). The 0.5 mg L^−1^ treatment group was the lowest concentration that suppressed bacterial growth below the MIC determination criterion as described in Materials and Methods. Accordingly, 0.5 mg L^−1^ was identified as the MIC_int_ of TGC against *Mab*. To further evaluate the bacterial killing effect of TGC under static conditions, the simulation period was extended to 7 days and higher dose groups up to 6.25 mg L^−1^ were explored as shown in Fig. 5B. From this second set of simulation results, TGC demonstrated a substantial bacterial killing effect against *Mab* even at a low static concentration level around MIC_int_.

With the standard dosing regimen of 50 mg every 12 h in humans, plasma exposure of TGC at steady state has been reported as 0.621 and 0.415 µg mL^−1^ for *C*_max_ and *C*_average_, respectively (24). Although TGC exhibits atypical non-linear plasma protein binding, it maintains approximately 35% unbound fraction within the range of clinically relevant exposures following IV infusion (27). Thus, the reported exposure metrics translate to 0.217 and 0.145 µg mL^−1^ of unbound TGC for *C*_max_ and *C*_average_, respectively. Given that the free fraction of TGC in 7H9 media remains constant at approximately 36% regardless of concentration as determined in our study (Fig. S4), it can be reasonably assumed that the number of drug molecules per unit volume contributing to bacterial killing is comparable between the two conditions. Therefore, the MIC_int_ determined by model-based simulations along with the bacterial killing profile at corresponding dose levels suggests that based on these plasma concentrations, TGC should exhibit a bacterial killing effect in patients with *Mab* infections.

The observed similarity of protein binding for TGC in human plasma and 7H9 media is not surprising as 7H9 with OADC contains albumin, and albumin has been described as the protein responsible for TGC plasma protein binding (28). Since MHB II lacks albumin and has a different composition from 7H9, potential differences in binding to media may have also contributed to the observed differences in MIC between MHB II and 7H9.

### Model-based characterization of determinants for *in vitro* susceptibility testing

**Fig 6:**
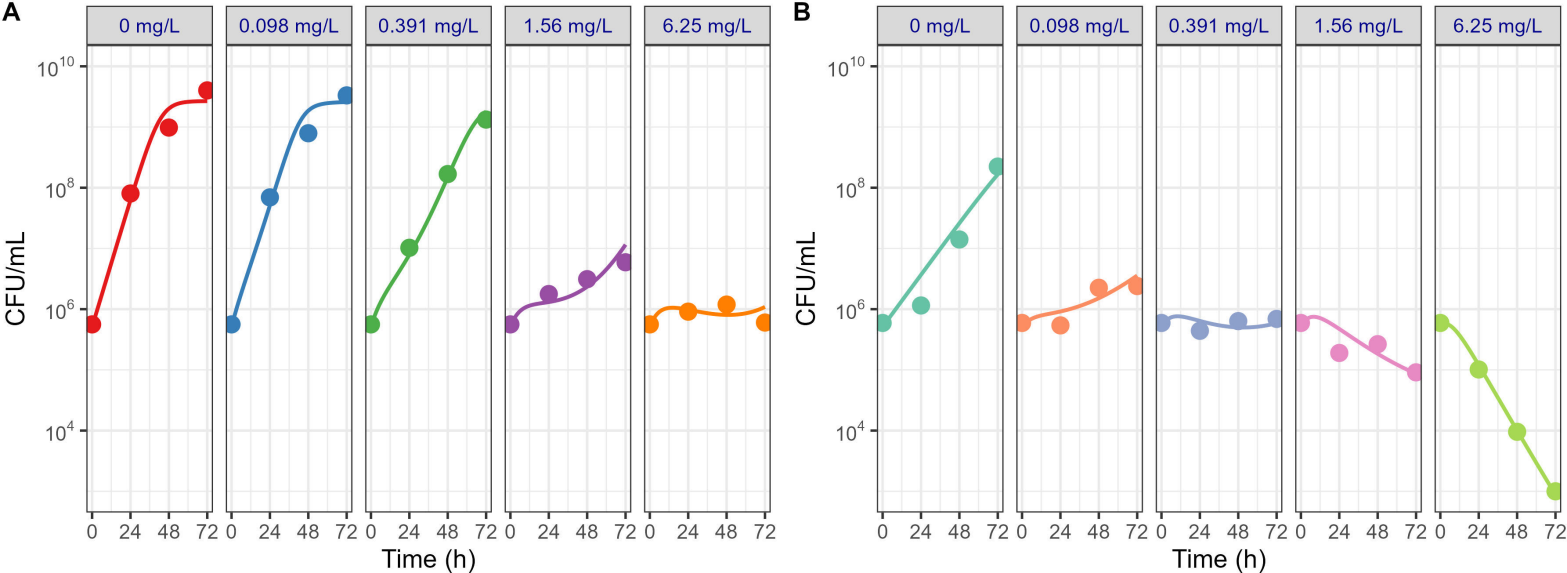
Observed (symbols) and model-based predicted (lines) of bacterial killing profiles during the *in vitro* susceptibility tests performed in 7H9 (**A**) and MHB II (**B**) media, respectively.

As differential TGC stability was observed in MHB II vs 7H9 media, the original model was modified to include covariates that describe differences in bacterial growth and drug stability in 7H9 and MHB II. The updated model was utilized to quantitatively assess the contribution of each factor influencing the bacterial killing efficacy of TGC against *Mab*. In this context, the robustness of the model and its applicability across various experimental conditions was confirmed. Updated model-based simulation profiles properly captured the bacterial killing effect of TGC assessed in different incubation media as depicted in Fig. 6. The corresponding parameter estimates, summarized in Table 3, indicated that the growth rate constant of *Mab* (*K*_0_) in MHB II (0.092 h^−1^) was 2.08-fold lower than in 7H9 (0.19 h^−1^). Inclusion of different degradation rate constants for TGC in 7H9 (0.044 h^−1^) and MHB II (0.017 h^−1^) measured in drug stability studies was adequately incorporated to account for experimentally observed differential bacterial killing effect of TGC against *Mab* in the respective media. Some model parameters, such as *K*_0_ of *Mab* and *K*_kill_._max_ of TGC, were similar for both media. However, KC_50_ of TGC against *Mab*, influenced by the modulation rate constant (α) describing the time-dependent change in susceptibility, was fivefold different presumably due to the short-term incubation period (72 h) and the limited number of observations which were insufficient to characterize the full extent of time-dependent susceptibility change of *Mab* to TGC as shown in the long-term microscale *in vitro* dynamic time-kill assay performed in the 7H9 media.

**TABLE 3:** Parameter estimates of PK/PD model used to characterize different pharmacological behaviors of TGC in 7H9 and MHB II

Parameter	Estimate	RSE*^c^* (%)
Fixed effects		
*V* (mL)	0.2	—^*a*^
*K*_deg.7H9_ (h^−1^)	0.044	—^*b*^
*K*_deg.MHB II_ (h^−1^)	0.017	—^*b*^
*N*_0_	5.11 × 10^5^	11.3
*K*_0_ (h^−1^)	0.19	11.3
Cov_*K*0_^*d*^	−0.73	18.7
*N*_max_	2.68 × 10^9^	20
*K*_kill.max_(h^−1^)	0.18	10.5
*KC*_50_ (µg mL^−1^)	0.11	41.8
β (h^−1^)^*e*^	0.16	32.3
Random effects		
ω [*K*_0_]	0.43	19.6
Residual error (proportional)		
*N*	0.47	5.43

^*a*^ Fixed on volume per well in 96-well plate.

^*b*^ Fixed value based on the results of TGC stability study in 7H9 and MHB II, respectively.

^*c*^ Relative standard error.

^*d*^ Covariate effect on parameter: if media is MHB II, $K_{0,MHB}=K_{0}\times e^{CovK_{0}}$.

^*e*^ Since the observation period was limited to 72 h, only β could be reliably estimated and α was omitted from the model.

## DISCUSSION

The treatment of NTM pulmonary infection requires at least 18 months according to an official ATS/IDSA statement (4). None of the currently recommended antibiotic regimens based on *in vitro* susceptibilities has consistently met their therapeutic goal for patients infected with *Mab* (15, 29). This observation underscores the strong need for accurate and reproducible drug susceptibility testing against NTM. However, as described in the present study, many antimicrobial drugs known to be efficacious against NTM species encounter stability issues in specific testing media, such as 7H9. Schoutrop et al. highlighted the stability concerns of NTM antimicrobial drugs, including rifampicin, clarithromycin, linezolid, amikacin, and cefoxitin in media, and their implications for drug susceptibility testing (18). Nevertheless, antibiotic exposure is usually assumed to be sustained and constant in traditional susceptibility testing, and antibiotic stability issues have been frequently disregarded.

The aim of the present study was to suggest an *in vitro* experimental system combined with a mechanism-based PK/PD modeling approach that can be used to overcome the methodological limitations of the existing standard MIC assessments. Our model-based MIC prediction indicated that the MIC_int_ of TGC was approximately 0.5 mg L^−1^, which is sevenfold lower than the widely reported MIC value measured under pseudo-static conditions (11, 12, 15, 16), demonstrating that clinically relevant exposure levels of TGC are sufficient to have a killing effect on *Mab*, thereby providing an explanation for the favorable therapeutic outcomes against *Mab* infections in clinical reports (7, 30, 31). Considering the reported higher TGC exposure in the lungs relative to plasma (32–35), this discussion becomes even more compelling.

The reliability of the model-based MIC_int_ prediction was supported by several factors. First, the model simultaneously accounted for single and multiple dose regimens, thereby representing diverse experimental conditions and inherent variability. Favorable diagnostic plots and low RSE of each model parameter further supported stability of the developed modeling framework. Additionally, as shown in Fig. 4, the model-based simulations conducted under *in vitro* experimental conditions for four different scenarios from the literature could reasonably recapitulate the corresponding bacterial elimination profiles in the respective papers. These results not only demonstrated methodological soundness but also underscored the robustness and predictive power of the modeling approach regarding the intrinsic antibacterial effect of TGC.

These experiments showed that TGC dissolved in MHB II was relatively stable, resulting in a fourfold lower MIC compared to TGC in 7H9 due to the higher level of sustained drug exposure. This finding suggests that the use of fresh MHB II can help partially mitigate TGC stability issues, bringing the MIC closer to the MIC_int_. However, the more than twofold lower doubling time of *Mab* in MHB II also needs to be considered. This effect on doubling time is because the relatively slower growth of *Mab*, a rapid-growing mycobacteria, in MHB II media can influence the determination of the MIC at the preselected observation point (72 h), which depends on the number of viable bacteria actively growing. The applied modeling approach appropriately captured the apparent pharmacological effect of TGC in the 96-well plate format for the *in vitro* susceptibility test, thereby accounting for the quantitative differences related to relative drug stability (2.58-fold) and bacterial growth rate (2.08-fold) and elucidated the distinct contributions of each parameter on their combined effects. These results further strengthen the validity of our modeling approach evaluating the intrinsic bacterial killing of TGC.

When the PK/PD index fAUC/MIC from our microscale *in vitro* dynamic time-kill assay system was compared to that obtained from a hollow fiber infection model (HFIM)-based dynamic time-kill assay in previously published literature, the PK/PD indices required for 1-log reduction were similar to each other (Fig. S5) (11). Considering that this publication also used the 7H9 media in their HFIM study, it can be concluded that the results of HFIM-based dynamic time-kill assay also reflected the limited stability of TGC in 7H9, although no detailed concentration-time data are provided. While our experimental system demonstrated complete eradication of *Mab* in the high-dose treatment group (Fig. 3), the HFIM study showed no complete eradication even after 21 days of consecutive treatment with an intermediate dose (11). The comparison of these results implies that the bacterial killing effect of TGC may be more effectively achieved with high doses even when administered as a single dose or over a short treatment period, rather than with the continuous exposure of low or intermediate TGC levels. This finding suggests that an efficient delivery method that achieves high exposure at the site of infection should be employed to maximize the bacterial killing effect (12, 15). Further validation of these *in vitro* results under *in vivo* conditions, however, is necessary to reliably assess the full potential of TGC in treating *Mab* infections.

Apart from the modeling approach, the *in vitro* experimental method we suggested here is noteworthy due to its relative simplicity, high throughput capability, and the fact that the assay results summarized based on the PK/PD index fAUC/MIC were comparable to those obtained from HFIM experiments. When evaluating the development of acquired resistance against TGC in *Mab* species, standardized *in vitro* static time-kill assays may result in inconclusive findings due to their pseudo-static condition of drug exposure. In contrast, our experiments allowed for performance under multiple dose conditions with drug replenishment and long observation periods, thereby providing a more robust assessment of potential resistance development. The regrowth of the *Mab* observed in our study still cannot be fully separated from the potential expression of resistance to TGC, but as presented, the primary driving force of the phenomenon seems to be drug degradation following last treatment at day 5 (or day 1 for the single dose), considering that timing of regrowth was closely correlated with the degradation rate of TGC in 7H9. For a definite conclusion regarding resistance development of *Mab* to TGC, however, a relatively longer treatment and observation period would be warranted. In addition, properties of closed experimental systems, which cannot overcome limited nutrient supply and accumulation of metabolic byproducts of *Mab*, need to be carefully considered when interpreting long-term efficacy and drug tolerance development.

In the clinical therapy of NTM, antimicrobials are frequently used as combination therapy. As the therapeutic benefits of TGC for the treatment of *Mab* infections are well recognized, it is necessary to determine how to maximize the clinical efficacy of TGC through combination therapies. For unstable drugs, evaluating intrinsic efficacy of drug combinations *in vitro* is challenging, and therefore, a supplement dosing strategy has been proposed (36, 37). Thus, *in vitro* experimental systems combined with mechanistic PK/PD modeling similar to the approach presented could provide a more relevant way to assess the antibacterial performance of combination regimens. Although the evaluation system suggested offers valuable insights into the intrinsic antibacterial activity of unstable antibiotics, the model does not incorporate the role of the host immune system in bacterial killing effect. Nevertheless, the exposure-response relationship and derived parameters such as KC_50_ and MIC_int_ can be considered critical building blocks for more sophisticated models to predict *in vivo* efficacy and clinical outcomes with higher accuracy across various therapeutic applications. Although the current study only investigated the *Mab* reference strain ATCC19977 and, thus, the generalizability of its findings may be limited, the same framework can be readily applied to other *Mab* strains and clinical isolates for broader interpretation.

In conclusion, based on the results presented, the potential therapeutic utility of TGC against pulmonary infections caused by *Mab* was confirmed and its potential applicability through the inhalation route to achieve maximum therapeutic benefit highlighted (12, 15). The *in vitro* experimental system, coupled with the PK/PD modeling framework, represents an accurate evaluation tool for unstable antimicrobials and may breathe new life into current therapeutic agents in terms of their clinical utility.

## SUPPLEMENTAL MATERIAL

Supplemental material aac.01025-25-s0001.pdf Table S1; Fig. S1 to S6.

ASM does not own the copyrights to Supplemental Material that may be linked to, or accessed through, an article. The authors have granted ASM a non-exclusive, world-wide license to publish the Supplemental Material files. Please contact the corresponding author directly for reuse.
