# Supplementary material for: In vitro PK/PD modeling and simulation to accurately assess the antimicrobial activity of tigecycline against Mycobacterium abscessus
Source: Antimicrob Agents Chemother. 2025 Dec 23;70(2):e01025-25. doi: 10.1128/aac.01025-25 (PMC12888882; doi:10.1128/aac.01025-25)
Supplement: Supplemental material — Table S1; Fig. S1 to S6. [file aac.01025-25-s0001.pdf]

Supplemental Material

To

In vitro PK/PD Modeling and Simulation to Accurately Assess the Antimicrobial Activity of Tigecycline Against *Mycobacterium abscessus*

Hyunseo Park, Sara E. Maloney Norcross, Anthony J. Hickey,

Mercedes Gonzalez-Juarrero, Bernd Meibohm

Table S1. Comparison of model selection criteria with and without inclusion of the time-dependent parameters alpha and beta

Criteria	α & β	α only	β only	No α & β
-2 x log-likelihood (OFV)	5597	5774	5688	5992
Akaike Information Criteria (AIC)	5623	5796	5710	6010
Bayesian Information Criteria (BIC)	5638	5809	5723	6020
Corrected Bayesian Information Criteria (BICc)	5672	5839	5753	6046

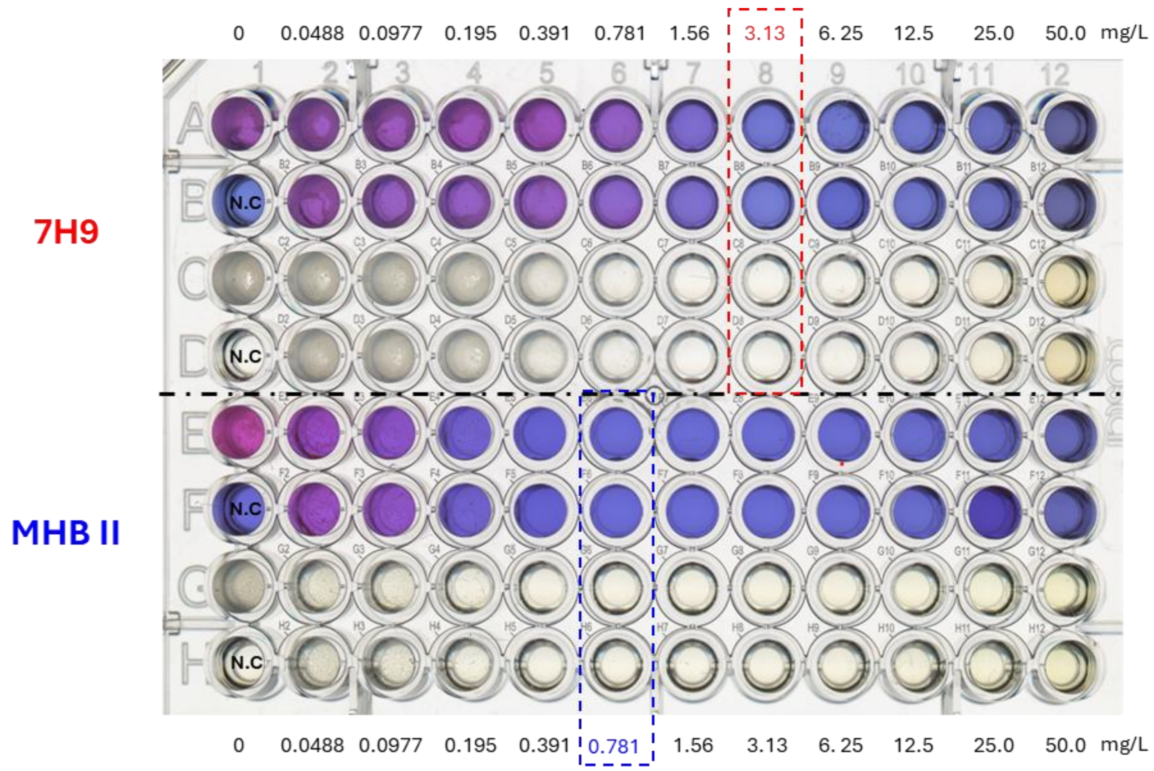

Figure S1. Results of susceptibility assay for TGC against *Mab*. MICs were determined using 96 well broth microdilution method in two different media 7H9 (red, top) and MHB II (blue, bottom). Final MIC in each media was determined based on high-resolution scanned image. N.C. represents negative control without *Mab* inoculation.

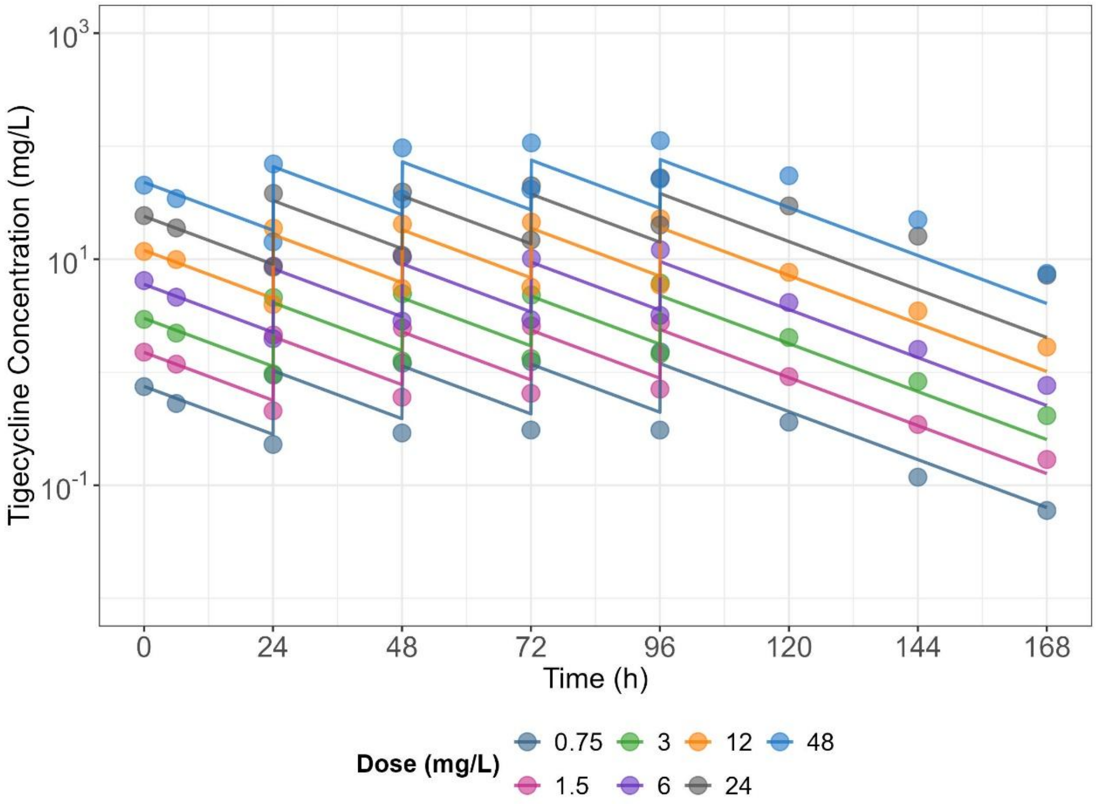

Figure S2. Longitudinal concentration profiles of TGC following multiple dose administration into 7H9 media in a 24-well plate. Circles indicate observed TGC concentrations, while solid lines represent theoretical values calculated using first-order degradation rate derived from experimentally determined half-life of TGC in 7H9. Daily dose expressed in mg/L, represents amount of TGC administered to each well per unit volume.

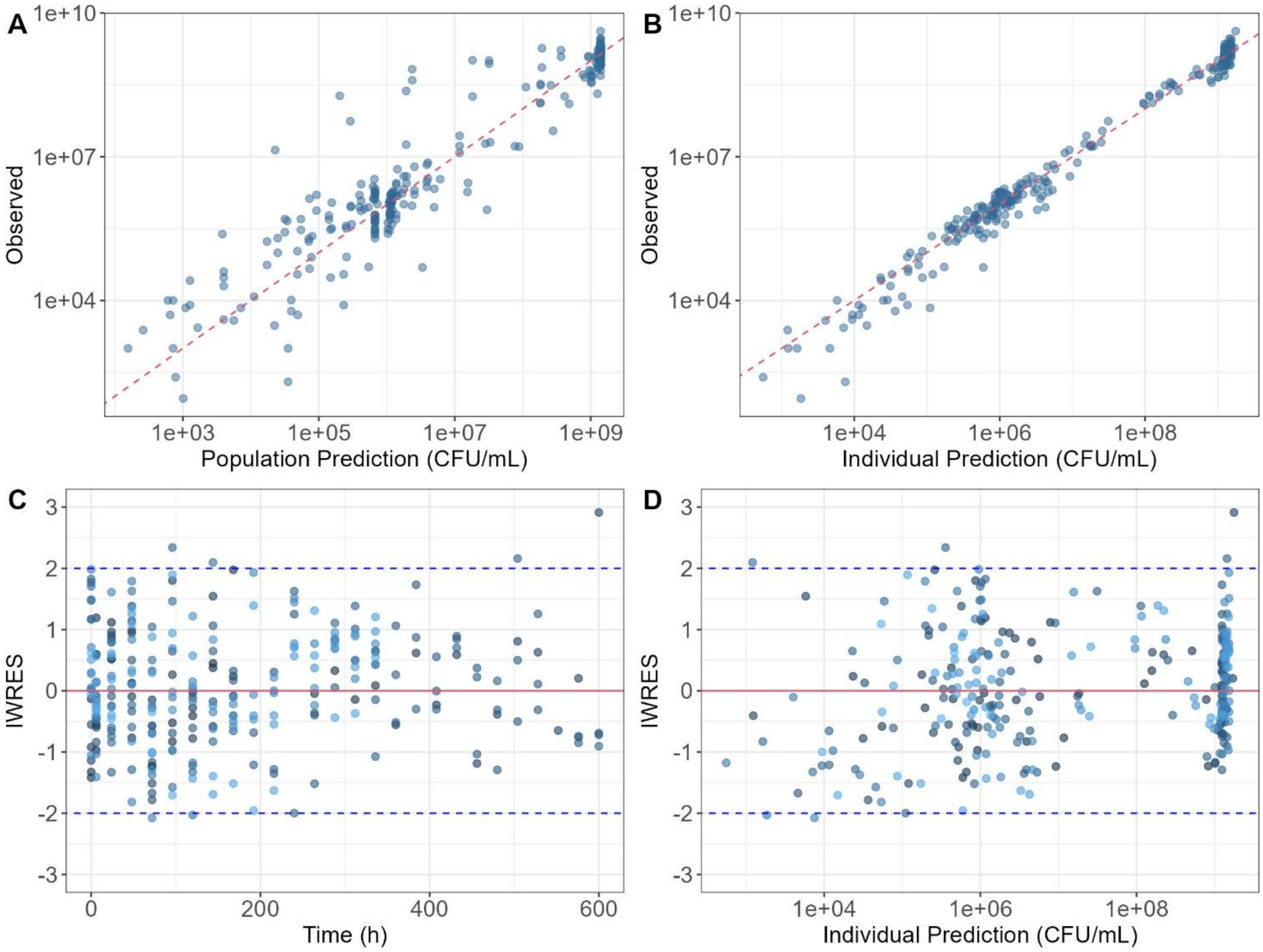

Figure S3. Diagnostic plots for *In vitro* PK/PD model.

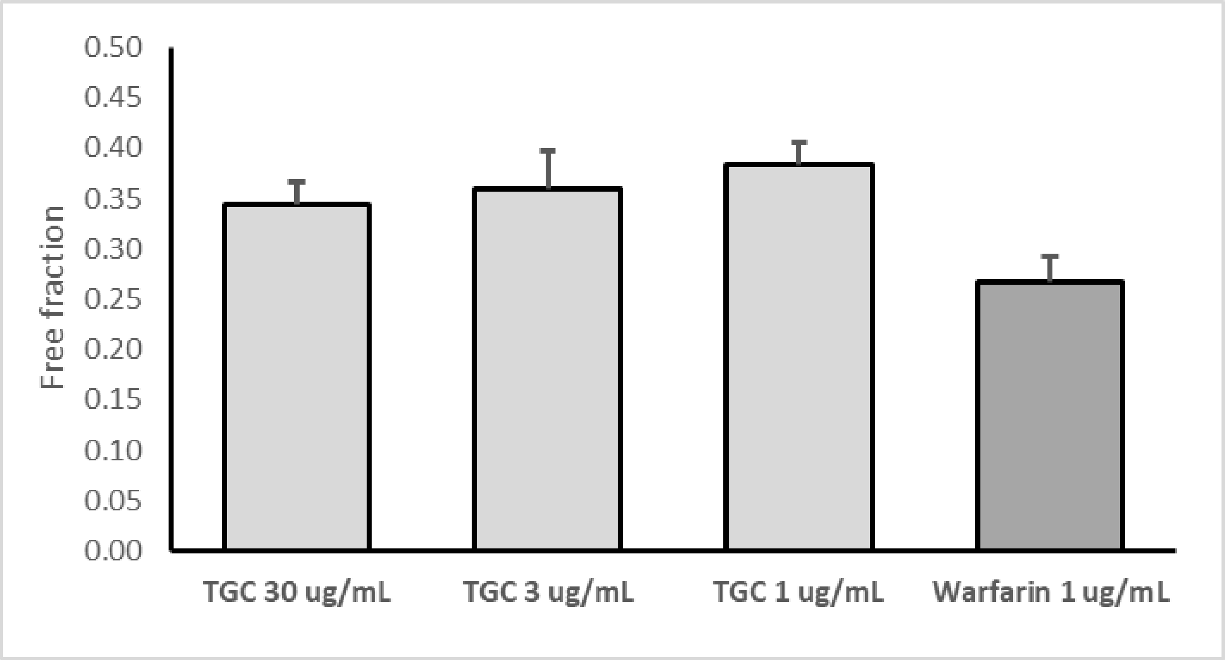

Figure S4. Protein binding of TGC at different concentrations in 7H9 media. Data are presented as mean ± standard deviation.

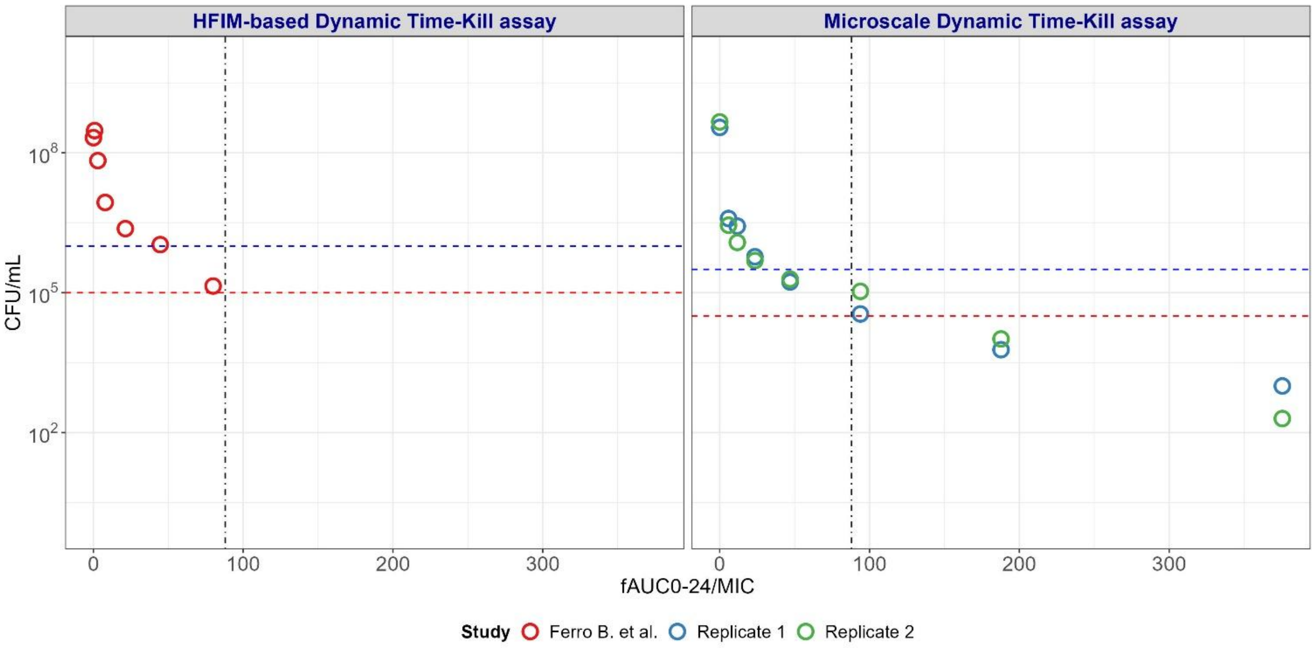

Figure S5. $fAUC_{0-24}$/MIC based PK/PD index occurring 1-log reduction. Blue and red dotted horizontal line indicate initial and 1-log reduced bacterial concentration, respectively. The PK/PD index required for 1-log reduction is presented in black dotted vertical line. HFIM-based dynamic time-kill assay result was digitized from

Ferro BE, Srivastava S, Deshpande D et al. Tigecycline Is Highly Efficacious against Mycobacterium abscessus Pulmonary Disease. *Antimicrob Agents Chemother* 2016; 60: 2895-900.

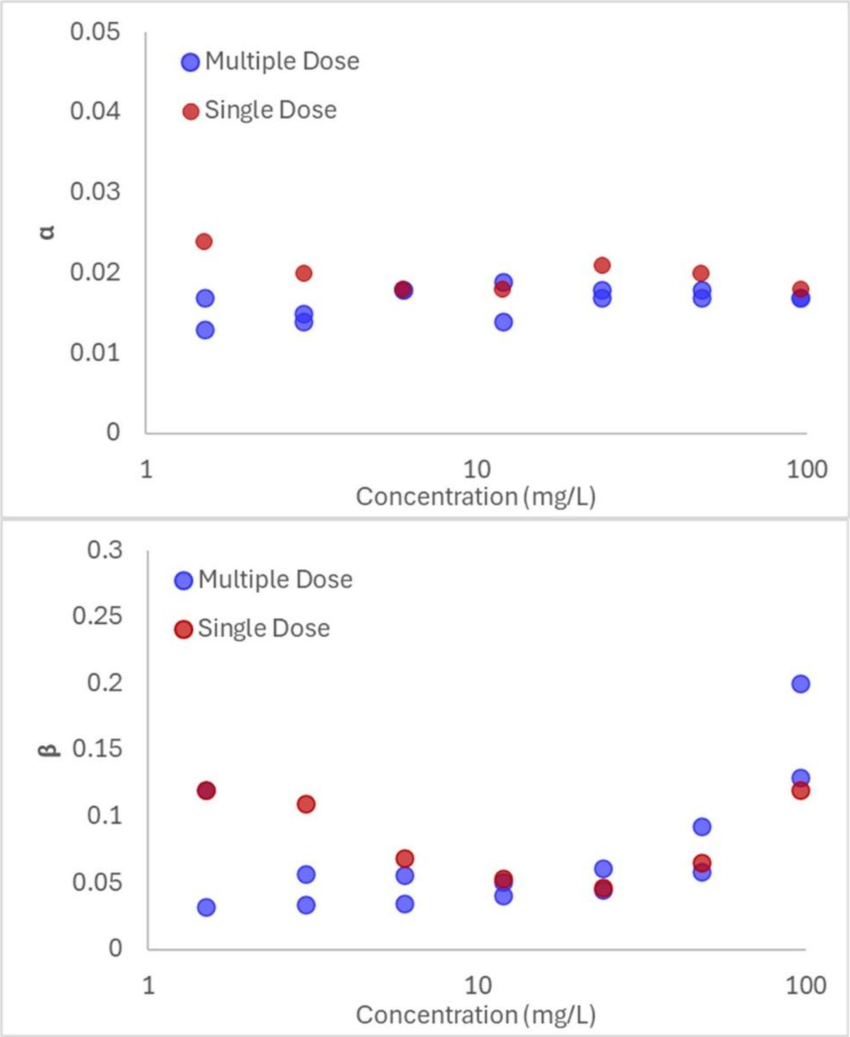

Figure S6. Obtained estimates for α and β versus incubation concentration under single- and multiple-dose conditions.
